# The War Investigation Commission

**Published:** 1898-11

**Authors:** 


Monthly Review of Medicine and Surgery.

EDITORS:

THOMAS LOTHROP, M. D. - - WM. WARREN POTTER, M. D.

All communications, whether of a literary or business nature, should be addressed to the managing editor: 284 FRANKLIN STREET, BUFFALO, N. Y.

VOL. XXXVIII. NOVEMBER, 1898. No. 4

### THE WAR INVESTIGATION COMMISSION.

PRESIDENT McKINLEY early in September appointed a commission composed of nine members to investigate the conduct of the war. This was done out of respect to a popular demand, created though it was, in whole or in part, by the clamor of the yellow newspapers, supported by unpatriotic sentiments. Of the first appointees several declined, but the commission as finally made up proved if anything stronger than at first constituted and is as follows: Major-general Grenville M. Dodge, of New York, chairman; Major-general Alexander McDowell McCook, U. S. Army (retired); Bvt. Major-general James A. Beaver, ex-Governor of Pennsylvania; Brigadier-general John M. Wilson, Chief of Engineers U. S. Army; Colonel Charles Denby, of Indiana; Colonel James A. Sexton, Commander-in-Chief, Grand Army of the Republic, of Illinois; ex-Governor Urban A. Woodbury, of Vermont; Captain Evan P. Howell, of the late Confederate Army, of Georgia; and Dr. Phineas S. Connor, formerly an assistant surgeon U. S. Army, of Cincinnati. Several of these men are lawyers, while General Beaver is a judge of the appellate court of Pennsylvania. He lost his right leg at Reams's Station, Va., August 25, 1864, and the amputation was of necessity made too high to permit the adjustment of an artificial limb. All of these men have rendered honorable, if not distinguished, service in the army of the United States, either regular or volunteer, except Captain Howell, and he is a veteran of the Confederate Army with an honorable record.

It will be seen that the personnel of the commission is above reproach and may be expected to make a thorough investigation of the general conduct of the war, as well as to make a report that will be both important and conclusive.

The President in defining the duties of the commission made the following comment:

> There has been, in many quarters, severe criticism of the conduct of the war with Spain. It is my earnest desire that you shall thoroughly investigate these charges and make the fullest examination of the administration of the war department in all its branches, with the view of establishing the truth or falsity of these accusations. I put upon you no limit to the scope of your investigation. I invite the closest scrutiny and examination and shall afford every facility for the most searching inquiry. The records of the war department and the assistance of its officers shall be subject to your call.

Nothing could have been clearer than the intention of the administration thus expressed, to make the investigation all that the word thorough implies. Nevertheless, unfriendly newspapers and hypercritical medical journals have given out, either by direction or implication, that the commission is engaged in a "white washing" enterprise. To so state either in words or by inuendo is to directly insult not only every member of the commission, but even the President himself. We think it high time that a halt was called in this whole business, and that these ochre-hued newspapers and jaundiced medical journals should be compelled to keep silence until the report of the commission is made public.

Thus far the commission has unearthed nothing that was not heretofore pretty well known, but the testimony it has taken has served to clear up many conditions in the military horison that were perhaps somewhat cloudy. For instance, when General Wheeler testified that the camp at Montauk was the finest temporary military camp the world had ever seen, and that the hospital was supplied with everything needed that money could buy, it settled those questions forever in the minds of every reasonable man. General Wheeler is a veteran of two wars, has seen as much field service as any American officer, and well knows war and its needs. No newspaper or politician can overturn the truths he evolved on the witness stand, which were told in that calm, simple manner, so characteristic of the man, that carried conviction to every listener or reader.

The star witness of the yellow newspaper party, Major Seaman, Surgeon of the 1st New York Engineers, who seemed impressed with the idea that he must pose as a reformer, and had apparently sailed from Porto Rico with that intent, was met and confounded at every point of his plaint. He has even been hoist with the petard of his own signature, and the officers of the departments of which he found fault have shown that he was at least mistaken in his charges.

The commission at the present writing is in Florida, taking testimony in regard to the camps in that state, and on every hand they have found affairs much better than represented. General Lee's corps in particular has been found to be in excellent condition, both as to supplies and health.

Several professional female nurses were heard at one of the Florida sessions, including Miss Copeland, chief nurse in the Third Division Hospital, Miss Robbins, head nurse in the First Division Hospital, Miss Hubbard, head nurse in the Second Division Hospital, and Misses Walker and Waters, ward nurses. Miss Copeland spoke in commendation of the conduct of the hospitals, and she said that the nurses were well treated and apparently greatly respected. There was in the beginning some scarcity of appliances for the sick, such as hot-water bottles, which, while not a necessity, were often a comfort. Some nurses had made complaints, but they were the kind of persons, she said, who are never satisfied.

This ought to be regarded as most convincing testimony, coming as it does from a source that knows the facts, and could not be charged with any disposition to pervert them.

We have felt impelled to make the foregoing comments on the work of the commission because of the fact that newspapers are still distorting the real conditions, and even some medical journals are lending their columns to similar purposes.

---

Brigadier-general Leonard Wood, U. S. V., the first Colonel of the Rough Riders, now military governor of Santiago de Cuba, is reflecting both credit upon the military and medical professions in his administration of the affairs of that city and province.

He has developed and put in force a sanitary code that has reduced the death-rate of Santiago more than 70 per cent. ; he has established police regulations that would do credit to any northern city ; he has reopened the public schools, in which he compels the teaching of English by competent instructors, who receive adequate salaries. In short, he has reëstablished civil government on a firm and liberal basis, even though it is administered by military power. General Wood is, as we have intimated, an ornament to the medical and military professions, and in his administration of affairs at Santiago has created a model that deserves imitation.

IN DISCUSSING the question of competence of army officers, especially as bearing upon the selection of camp sites and the responsibility therefor, the *New York Tribune* some time ago made the following pertinent editorial comment:

So it has been easy to say that the staff has been weakened by adding to it men from civil life. But what would the critics have? Congress permitted an army of but 25,000 men and barely a staff sufficient for that number. Suddenly this army had to be expanded to 250,000 men. Could the old staff be spread over an army ten times as large? If not, what recourse was there, excepting to make the old staff go as far as it could and eke out with appointments of the best men that could suddenly be found in the militia or from civil life? And if the result was often not satisfactory, who was most to blame—the Congress that refused to provide for a larger staff in time of peace, or the authorities who did the best they could with the material at hand when war came?

---

QUININE is a drug of the utmost importance in the treatment of many diseases, and the war between the United States and Spain, occurring as it has in the tropics, tends to its increased use. As bearing upon this subject a recent communication from Sidney B. Everett, United States Consul at Batavia, Island of Java, possesses special interest.

Writing under date of June 17th, he says that just now things look especially bright for the industry, as the war has resulted in a rise in quinine quotations. For some years Java has been the principal country for the production of cinchona bark. Yet the actual manufacture of sulphate of quinine there has only just begun. The first shipment of record was in last January, and was one of 10,000 ounces. It was shipped to the United States. Up to the date of the report there has been invoiced and shipped to this country 48,300 ounces of sulphate of quinine, valued at $11,395.55 Mr. Everett believes that more will follow.

The success of the one factory in existence seems probable. This factory, the paper accompanying the consul's report shows, buys no bark, but works on commission, the actual proceeds of the finished product going to the planter. It is urged that now is the time to begin new quinine enterprise. The stock now laid up will, it is stated, be consumed in two or three years, and the demand will immediately be largely increased. The quinine made by the American houses comes from bark which is first sent from Java to Amsterdam for sale and then shipped over to the United States

Mr. Everett's authority, F. W. Sijthoff, manager of the Javanese factory, claims that Java can produce sufficient raw quinine to supply America, making " that country entirely independent of Europe," to quote his language. He mentions America first in the list of countries supplied by the Java factory. Consul Everett says he cannot conceive of a better investment than the planting of cinchona in Java. Concession of lands are not hard to get there, if one is on the spot. The climate in the interior is as perfect as that of the coast cities is bad. The Dutch government has made a great success of the planting, clearing a profit in 1896 of over $38,500 from its estates. The world's output is only 788,771 pounds, of which Java produces about three-fourths. In 1897 North America imported in cinchona bark and quinine 10,000,000 ounces of sulphate of quinine.

---

A RECENT dispatch from Washington states that an order has been issued by the War Department directing the Surgeon-general to convene a board of medical officers to examine acting assistant surgeons now in the service as well as candidates for appointments. This is as it should be.

During the progress of the war the demand for medical officers was so great and the immediate necessity so imperative that it was found impracticable to establish or enforce this rule. Now, however, the emergency is past and the surgeon-general does wisely in protecting his department against the liability of incompetent men, by subjecting applicants to adequate examination to determine their fitness to serve as medical officers.

---

COLONEL L. M. MAUS, chief surgeon of the 7th Army Corps, commanded by General Fitzhugh Lee, at Jacksonville, Fla., has recently made a most elaborate report relating to the health of that command. Colonel Maus has had twenty-four years' experience in the regular army and his opinions, as expressed in his report to the war department, are entitled to the most patient consideration. After reciting the whole situation in detail he concludes his report with the following recommendations :

1. That the medical officers of the national guard be organised in a separate corps, like that of the medical department of the United States Army, and that all such officers be required to pass a rigid examination before receiving a commission before a board, consisting of one medical officer of the United States Army, and two of the national guard ; and that they be required to report once yearly at the national guard camps for instruction in professional and administrative work, under the guidance of a medical officer of the

United States Army, detailed by the war department, who shall likewise have authority to call upon these medical officers at anytime during the year for reports as regards the work they are doing in their military organisations. That these medical officers of the national guard be supplied with all of the necessary blanks, books, and the like, arranged for the administration of the medical department by the Surgeon-general of the army.

2. That the hospital corps of the national guard be organised the same as that of the United States Army and independent of regiments. That in time of peace this corps will consist of 3 per cent. of the strength of the national guard, and in time of war of 5 per cent. That the men be required to assemble once yearly at the camps of the national guard for instruction in litter drill, first aids, nursing, care of the sick, and preparation of food, under the instruction of a medical officer of the United States Army, detailed by the war department, and that acting stewards and hospital stewards be appointed from these men after a professional examination before a board of medical officers.

3. That division surgeons be required to pass an examination before a board of three medical officers as to their competency to administer the medical department of a division before receiving commissions, unless they are detailed from the medical department of the United States Army. That military surgeons of known experience in military matters be specially designated for this purpose.

4. That brigade surgeons shall also be required to pass an examination on administrative and professional work before they can receive commissions.

5. That regimental surgeons be abolished, and that the medical department be organised as indicated above under remarks of medical officers.

6. That ample provision be made for hospital tents, constructed of papier maché, and so arranged as they can be shipped and set up at any point where needed. That these tents be supplied with floors, proper windows, doors and ventilation, and so arranged that they can be attached together and form wards.

7. That all hospital cots supplied to the United States Army be wire-woven, with iron or steel frames.

8. That the national guard of the United States be under the control of the national government, as far as summer camps are concerned, and that the expense of these summer camps be defrayed by the national government, and that every officer and soldier of the national guard be placed upon a payroll by the national government for the time he is actually in service. That uniforms for the enlisted men of the national guard be paid for by the national government.

---

The following paragraph taken from a daily newspaper (we are sorry not to be able to give its name) contains wholesome suggestions which deserve to be pondered, especially by business men :

" The influence of fatigue on digestion is pretty well understood. Scientific experiments have demonstrated the fact beyond a doubt. They have even gone further and shown that fatigue is a disease, and that it is possible to produce the same symptoms in one animal organisation by innoculation with the fatigued serum of another, showing that overwork produces an actual poison in the system. Worry is equally antagonistic to good digestion, another fact that is well known, but cannot be too often reiterated to this nation of worrying folk. A little rest and banishment of care in preparation for a meal should become a habit It means lengthened life and preserved health, as do such other confessedly hygienic habits as proper bathing, dressing and wholesome food."

---

We devote considerable space in this issue to the publication of an extended abstract from the report of the Surgeon-general of the United States Navy. It is a document of great interest and will well repay the time devoted to its reading.

---

In view of the fact that the patent office at Washington has issued a patent on diphtheria antitoxin to a foreign inventor or manufacturer, it is gratifying to note the determination of Messrs. Parke, Davis & Co., of Detroit, to contest its validity in the courts. This eminent house also proposes, pending a decision, to protect and defend at its own expense physicians in any legal proceedings that may be brought as a result of purchase or use of the antidiphtheritic serum that P., D. & Co. manufacture and offer for sale. It is about time that American physicians were freed from the incubus of foreign patents upon therapeutic resources.

